# Agreement between neuroimages and reports for natural language processing-based detection of silent brain infarcts and white matter disease

**DOI:** 10.1186/s12883-021-02221-9

**Published:** 2021-05-11

**Authors:** Lester Y. Leung, Sunyang Fu, Patrick H. Luetmer, David F. Kallmes, Neel Madan, Gene Weinstein, Vance T. Lehman, Charlotte H. Rydberg, Jason Nelson, Hongfang Liu, David M. Kent

**Affiliations:** 1 Department of Neurology, Tufts Medical Center, Box 314, 800 Washington Street, Boston, MA 02111 USA; 2 Department of Health Sciences Research, Mayo Clinic, Rochester, MN USA; 3 Department of Radiology, Mayo Clinic, Rochester, MN USA; 4 Department of Radiology, Tufts Medical Center, Boston, MA USA; 5 Institute for Clinical Research and Health Policy Studies, Tufts Medical Center, Boston, MA USA

**Keywords:** Artificial intelligence, Silent brain infarct, White matter disease, Leukoaraiosis, Imaging

## Abstract

**Background:**

There are numerous barriers to identifying patients with silent brain infarcts (SBIs) and white matter disease (WMD) in routine clinical care. A natural language processing (NLP) algorithm may identify patients from neuroimaging reports, but it is unclear if these reports contain reliable information on these findings.

**Methods:**

Four radiology residents reviewed 1000 neuroimaging reports (RI) of patients age > 50 years without clinical histories of stroke, TIA, or dementia for the presence, acuity, and location of SBIs, and the presence and severity of WMD. Four neuroradiologists directly reviewed a subsample of 182 images (DR). An NLP algorithm was developed to identify findings in reports. We assessed interrater reliability for DR and RI, and agreement between these two and with NLP.

**Results:**

For DR, interrater reliability was moderate for the presence of SBIs (*k* = 0.58, 95 % CI 0.46–0.69) and WMD (*k* = 0.49, 95 % CI 0.35–0.63), and moderate to substantial for characteristics of SBI and WMD. Agreement between DR and RI was substantial for the presence of SBIs and WMD, and fair to substantial for characteristics of SBIs and WMD. Agreement between NLP and DR was substantial for the presence of SBIs (*k* = 0.64, 95 % CI 0.53–0.76) and moderate (*k* = 0.52, 95 % CI 0.39–0.65) for the presence of WMD.

**Conclusions:**

Neuroimaging reports in routine care capture the presence of SBIs and WMD. An NLP can identify these findings (comparable to direct imaging review) and can likely be used for cohort identification.

**Supplementary Information:**

The online version contains supplementary material available at 10.1186/s12883-021-02221-9.

## Introduction

Silent brain infarcts (SBIs) and white matter disease (WMD) present a conundrum in clinical practice and research. These silent cerebrovascular diseases are common in older adults and are associated with future risk of symptomatic stroke and dementia [1–3]. However, both are detected incidentally when neuroimaging is obtained for unrelated indications. The absence of symptoms, ICD codes for SBIs, or broad recognition of their significance impedes research to advance their diagnosis and the prevention of stroke and dementia after detection [4]. Accordingly, there are no clinical trials or large comparative effectiveness studies guiding strategies for management of most SBIs and WMD in adults [5].

Patient identification with artificial intelligence (AI) offers promising approaches for cohort development in light of these barriers. While AI can analyze data in various forms (including images and text), natural language processing (NLP) algorithms assessing neuroimaging report text is a pragmatic approach [6]. However, despite the development of consensus radiological definitions for SBIs and WMD, it is unclear how reliably these findings are reported in routine care [7]. Accordingly, it is uncertain whether an NLP algorithm can identify SBIs and WMD from neuroimaging reports in agreement with a neuroradiologist reviewing the neuroimages directly. To assess the feasibility of using a text-based AI to identify patients from electronic health records (EHRs), we assessed agreement between neuroimages directly reviewed by neuroradiologists and an NLP algorithm, using report interpretation by radiologists as a link.

## Methods

### Design and sample

Agreement was assessed between direct review (DR) by neuroradiologists (blinded to reports), report interpretation (RI) by radiology residents (blinded to neuroimages), and identification by an NLP algorithm. Patients older than 50 years with neuroimaging (CT, MRI) between 1/2009 and 10/2015 and no history of stroke, transient ischemic attack (TIA), or dementia were identified at two centers (Mayo Clinic, Tufts Medical Center). 1000 CT and MRI studies (500 each) were randomly selected through a previously described protocol [8]. The residents and neuroradiologists were instructed on annotation of the presence and characteristics of SBIs and WMD according to a consensus guide developed by two study investigators (LYL, PHL) (Additional file 1: Expanded Methods). Each resident and neuroradiologist completed an initial training set. Four residents (two from each center) performed RI on 1000 reports. A random subset of 400 reports were doubly read to assess interrater reliability. An initial NLP was developed to identify limited findings (SBI presence, WMD presence) in the 1000 reports (see Additional file 1: Expanded Methods). Another subset of 182 neuroimages (a number determined through an iterative attempt to obtain a stratified, random sample based on site, neuroimaging modality, and SBI presence) was directly reviewed and doubly read by four neuroradiologists (two from each center).

### Covariates

Data was collected on the age and sex of participants, scan modality, and scan year. SBI characteristics included presence, acuity (acute/subacute, chronic, both, not specified), location (lacunar/subcortical, cortical/juxtacortical, both, not specified), and number (one, two or more, not specified). WMD characteristics included presence and WMD grade (five level scale for RI, ten level scale based on the Manolio scale for DR) [9]. All collected data were reviewed for completeness.

### Statistical analysis

Interrater reliability between pairs of report annotators and neuroimaging readers for RI and DR and agreement between RI, DR, and NLP were assessed with statistical tests fitting the structure of the data (Cohen’s Unweighted Kappa for SBI and WMD presence, SBI acuity, SBI location; and Spearman rank correlations for SBI number and WMD grade) with these rankings: <0.00 poor, 0.00-0.20 slight, 0.21–0.40 fair, 0.41–0.60 moderate, 0.61–0.80 substantial, 0.81-1.00 almost perfect. Statistical analyses were performed in RStudio version 1.2.5033 as complete case analyses.

### Data availability statement

The data supporting this study’s findings (neuroimaging report text, NLP algorithm) are available from the corresponding author upon reasonable request.

## Results

### Cohort characteristics

Characteristics of the cohort were described previously [8]. The mean ages were 65 (± 10.6) and 66 (± 9.7) for the Mayo and Tufts cohorts. Women represented 48.6 and 54.8 % of the two cohorts. Prevalence of SBIs and WMD were 11.4 and 58.2 % for Mayo and 7.6 and 52.8 % for Tufts.

### Interrater reliability for report interpretation: link between direct review and NLP

For RI, interrater reliability was almost perfect for most findings including SBI presence, SBI number, WMD presence, and WMD grade (Supplemental Table 1). RI interrater reliability was substantial for SBI acuity and fair for SBI location.

### Interrater reliability for direct review: benchmark for NLP performance

For DR, interrater reliability was moderate for the SBI presence (*k* = 0.58, 95 % CI 0.46–0.69) and SBI location, and it was substantial for SBI acuity and number (Supplemental Table 1). Interrater reliability was moderate for WMD presence (*k* = 0.49, 95 % CI 0.35–0.63) and substantial for WMD grade. Interrater reliability was similar between CT and MRI for both SBI and WMD presence (Supplemental Table 2). Intra-institution and inter-institution interrater reliability were also similar (Supplemental Table 3).

### Agreement between report interpretation and direct review

Overall, data from routine care neuroimaging obtained by radiologists reading reports were in fair to substantial agreement with that obtained from direct imaging review (Table 1). Agreement was substantial for the SBI presence, SBI number, and chronic SBIs, and it was moderate for acute SBIs. Regarding SBI location, agreement was substantial for subcortical localizations, but it was only fair for cortical localizations. Agreement was substantial for the WMD presence and moderate for WMD grade.

**Table 1:** Agreement for SBIs and WMD

Characteristic	Cohen’s kappa or Spearman’s	95 % CI	*p*
DR with RI (*n* = 182)			
± SBI	0.77	0.67–0.86	
SBI acuity: acute/subacute	0.59	0.23–0.95	
SBI acuity: chronic	0.64	0.53–0.76	
SBI number	0.76		< 0.001
SBI location: subcortical	0.63	0.50–0.76	
SBI location: cortical	0.35	0.17–0.53	
± WMD	0.65	0.54–0.77	
WMD grade	0.60		< 0.001
NLP with RI (*n* = 1000)			
± SBI	0.86	0.78–0.94	
± WMD	0.85	0.77–0.83	
NLP with DR (*n* = 182)			
± SBI	0.64	0.53–0.76	
± WMD	0.52	0.39–0.65	

NB: *p*-values are only calculated for Spearman’s rank-order correlation

### Agreement of NLP with report interpretation and direct review: NLP performance

The identification of the presence of SBIs and WMD by NLP was in almost perfect agreement with that obtained from the same reports by radiologists (Table 1). The major disagreements between RI and NLP can be summarized as (1) rare expressions that were unseen during the development of NLP system, (2) complex sentence structure such as expressions requiring coreference resolution, and (3) human errors during the interpretation of neuroimaging reports. For example, the following excerpt from a neuroimaging report involves complex sentence structure that requires the NLP to comprehend the findings from multiple individual sentences and understand that the “findings” were referring to the above mentions in order to make correct prediction of the presence of an SBI: “Scattered, nonspecific T2 foci, most prominently in the left parietal white matter where there is an associated region of nonenhancing encephalomalacia and linear hemosiderin disposition. Linear hemosiderin deposition overlying the right temporal lobe (series 9, image 16) as well. No abnormal enhancement today. The above findings are nonspecific but the evolution, hemosiderin deposition, and gliosis suggest post ischemic change.” In a few cases, the NLP correctly ascertained the presence of SBI or WMD which was missed by human readers, demonstrating the consistency and high throughput of system augmented information extraction.

 Agreement between the NLP and the direct review by neuroradiologists of the same neuroimages was substantial for the SBI presence (*k* = 0.64, 95 % CI 0.53–0.76) and moderate for WMD presence (*k* = 0.52, 0.39–0.65). Additional performance measures of the NLP as compared to DR (F1-score, precision, recall) are reported in Table 2. The majority of cases where RI and NLP disagreed with DR were due to missing documentation of the incidental findings during the initial imaging interpretation.

**Table 2:** Direct Comparison Between NLP and DR for SBIs and WMD

NLP vs. DR	F1-score	Precision	Recall
SBI (*n* = 182)	0.773	0.797	0.750
WMD (*n* = 182)	0.841	0.896	0.792

## Discussion

In this study, identification of SBIs and WMD in routine care neuroimaging reports by an NLP algorithm corresponded with identification of these findings by direct review of the neuroimages. The level of agreement between NLP and DR was comparable to the benchmark of interrater agreement of two neuroradiologists following a research protocol to identify SBIs and WMD. Notably, interrater agreement of DR was moderate for the presence of SBI or WMD, highlighting the challenges neuroradiologists encounter in classifying these lesions, even when adhering to a strict research protocol. Nonetheless, these findings suggest that it is feasible to identify patients with SBIs and WMD for clinical studies using an AI-based cohort development strategy in EHRs.

There was also considerable agreement between granular data obtained from reports and neuroimages regarding characteristics of SBIs (acuity, location, number) and WMD grade which may be identifiable by a revised NLP and may help with stratification in future studies. SBIs are likely heterogeneous in acuity, mechanism, and risk of future stroke. Radiological characteristics offer insight on likely mechanisms of infarction that may warrant targeted prevention therapies. For example, SBIs with subcortical locations are likely related to hypertension as opposed to cortical SBIs which are likely due to embolism [10]. WMD severity is well-established to be associated with increased risk of dementia and progression of cognitive decline [3].

Regarding strengths, the collaboration of two referral centers increased diversity of the population and heterogeneity of neuroimaging interpretation and language. One limitation is that the centers are both academic: the radiology interpretation practices and language may not be generalizable to non-academic centers. Another limitation is that this study did not include a qualitative analysis illustrating the extent and detail to which SBI and WMD findings are present in neuroimaging reports. Finally, an additional limitation is that the NLP algorithm was designed to only assess the presence of SBIs and WMD. Nonetheless, the current algorithm is pragmatic and sufficient to identify patients who can undergo a more detailed clinicoradiological review for inclusion in clinical studies. Future research may include refinement of the NLP to assess granular features of SBI (e.g. acuity, location, number) and WMD (grade), and it may include additional NLP annotators (e.g. machine learning) to improve system performance generalizability and avoidance of overfitting.

## Conclusions

Neuroimaging reports obtained in routine care capture the presence of SBIs and WMD. An NLP algorithm identifying these findings can facilitate cohort development for clinical studies of patients with SBIs and WMD for prevention of future stroke and dementia.

## Supplementary information

**Additional file 1: **Expanded Methods. **Supplemental Table 1. **Interrater reliability for SBIs and WMD for RI and DR. **Supplemental Table 2.** Interrater Reliability for DR Across CT and MRI. **Supplemental Table 3.** Interrater Reliability for DR Across Two Institutions.

## Data Availability

The datasets used and analyzed during the current study available from the corresponding author on reasonable request.

## Abbreviations

AI: Artificial intelligence
CT: Computed tomography
DR: Direct review
EHR: Electronic health records
ICD: International Classification of Diseases
LYL: Lester Y. Leung
MRI: Magnetic resonance imaging
NLP: Natural language processing
PHL: Patrick H. Luetmer
RI: Report interpretation
SBIs: Silent brain infarcts
TIA: Transient ischemic attack.
WMD: White matter disease
