# Supplementary material for: Agreement between neuroimages and reports for natural language processing-based detection of silent brain infarcts and white matter disease
Source: BMC Neurol. 2021 May 11;21:189. doi: 10.1186/s12883-021-02221-9 (PMC8111708; doi:10.1186/s12883-021-02221-9)
Supplement: Supplementary file 1 — Additional file 1: Expanded Methods. Supplemental Table 1. Interrater reliability for SBIs and WMD for RI and DR. Supplemental Table 2. Interrater Reliability for DR Across CT and MRI. Supplemental Table 3. Interrater Reliability for DR Across Two Institutions. [file 12883_2021_2221_MOESM1_ESM.docx]

**Agreement between neuroimages and reports for natural language processing-based detection of silent brain infarcts and white matter disease**

Lester Y. Leung, MD, MSc, Sunyang Fu, MHI, Patrick H. Luetmer, MD, David F. Kallmes, MD, Neel Madan, MD, Gene Weinstein, MD, Vance T. Lehman, MD, Charlotte H. Rydberg, MD, Jason Nelson, MPH, Hongfang Liu, PhD, David M. Kent, MD, MS

**Supplemental Appendix**

**Expanded Methods**

*Development of the NLP algorithm*

Using the dataset of 1000 randomly selected neuroimaging reports from both medical centers, two NLP algorithms were developed to extract findings on SBIs and WMD from neuroimaging report text. Their performance was compared to identify the optimal AI-based strategy for identification of SBI and WMD findings. The algorithms adopted the open source NLP pipeline MedTagger for generic NLP processing (sentence tokenization, text segmentation, and context detection) and task-specific knowledge engineering (coding of specific words and phrases referencing SBIs and WMD). A summarization component assigned labels to the report text by applying heuristic rules. Domain-specific NLP knowledge engineering involved prototype algorithm development (using pointwise mutual information to identify words or language patterns referring to SBIs and WMD), formative algorithm development on a training data set, and final algorithm evaluation on a blinded test data set. False classifications were reviewed by two investigators (LYL, PHL) with subsequent iterative modifications to the keywords used by the NLP algorithm. The final refined algorithm was selected for comparison against RI and DR.[6]

**Expanded Methods: Neuroimaging report interpretation annotation guide**

Template hierarchy

1. SBI_found
   1. Acuity
      1. acute/subacute
      2. chronic
      3. both
      4. not specified
   2. Location1
      1. lacunar/subcortical
      2. cortical/juxtacortical
      3. both
      4. not specified
   3. Location2
      1. frontal
      2. parietal
      3. temporal
      4. occipital
      5. insular
      6. basal ganglia
      7. caudate
      8. putamen
      9. globus pallidus
      10. thalamus
      11. brainstem
      12. cerebellum
      13. more than one
      14. not specified
   4. Number
      1. one
      2. two or more
      3. not specified
   5. Negation
      1. yes
      2. no
   6. Comment
2. SBI_indeterminate (use for indeterminate findings, such as “possible” SBI or WMD lesions)
   1. Type
      1. encephalomalacia
      2. perivascular space
      3. possible lacune (lacunar infarct)
      4. other
   2. Negation
      1. yes
      2. no
3. WMD_found
   1. Grade
      1. mild
      2. mild/moderate
      3. moderate
      4. moderate/severe
      5. severe
      6. no mention of quantification
   2. Negation
      1. yes
      2. no

**Basic guidelines**

1. Markup text for the same neuroimaging finding (i.e. SBI, WMD lesion) in **both** the body (Findings) and summary (Impression, Assessment) sections of the report. Highlight them **separately** (the annotations may not match up perfectly if the reporting of findings is inconsistent).
2. If a field is left blank, that implies “not specified.”
3. “Negation” refers to the presence of a negative statement indicating the absence of a finding (i.e. a double negative).
   1. Example: “There is no evidence of acute infarction” would be annotated as SBI_found -> Acuity/Acute + Negation/Yes
4. If there is no mention of terms or synonyms suggesting infarction or white matter disease, do not annotate anything. Save the report as an XML to indicate that the report was reviewed.
5. Redacted, corrected, or “strike through” text in the neuroimaging reports should not be annotated.

**Language related to brain infarcts**

| **Category** | **Term** | **Suggested Category** | Example |
| --- | --- | --- | --- |
| Synonyms for infarct | Foci of restricted diffusion, restricted water diffusion, restricted diffusivity, diffusion restriction | Negation -> No  (i.e. infarct present) | “No focal masses, focal atrophy, or foci of restricted water diffusion.”  “No restricted diffusion.” |
| Certainty of infarct | Probable, likely | Negation -> No | T505: “which is likely secondary to a subacute infarct.” |
|  | Possible (or ambiguous language) | Annotate under SBI_indeterminate | “Prominent perivascular space or chronic lacunar infarct” |
|  | Encephalomalacia (not otherwise specified) | Annotate under SBI_indeterminate | T555: “Loss of gray-white matter differentiation and volume loss within the left middle frontal gyrus is consistent with an area of encephalomalacia.” (No subsequent mention of the nature of this lesion.) |
| Location of infarct | Lacune | Subcortical | T513: “There are old lacunes in the bilateral basal ganglia…” |
|  | Gyrus, cortex | Cortical | T581: “Small old infarcts are seen in the pre-central gyrus of the right frontal lobe, right parietal cortex and right occipital cortex.” |
| Number of infarcts | Several | 2 or more | T518: “Several foci in the cerebellum, right more than left also reflect old infarcts.” |

**Language related to white matter disease/leukoaraiosis**

| **Category** | **Term** | **Suggested Category** | Example |
| --- | --- | --- | --- |
| Synonyms for white matter disease | Chronic microangiopathy | Negation -> No  (i.e. WMD present) |  |
|  | Chronic small vessel ischemic disease | Negation -> No |  |
|  | Chronic microvascular ischemic disease | Negation -> No |  |
|  | Leukoaraiosis | Negation -> No |  |
|  | Periventricular white matter T2/FLAIR hyperintensity, likely representing chronic microvascular ischemic disease | Negation -> No |  |
|  | Chronic ischemic and/or degenerative changes | Negation -> No |  |
|  | Senescent white matter changes | Negation -> No |  |
|  | Nonspecific T2/FLAIR hyperintensity | Negation -> No |  |
|  | White matter hypodensity or T2 hyperintensity **without a differential** | Negation -> No |  |
|  | White matter hypodensity or T2 hyperintensity **with a long differential and no commitment to a “likely” diagnosis** | If microvascular or ischemic disease is mentioned -> Negation -> No  If microvascular or ischemic disease is not mentioned -> Do not annotate |  |
|  | White matter hypodensity or T2 hyperintensity **with a short differential mentioning *chronic microvascular ischemic disease* and *migraines*** | Negation -> No |  |
| Quantification of white matter hyperintensities (MRI) or hypodensities (CT) | Few | Mild | T531: “There are a few scattered nonspecific foci of T2/FLAIR hyperintensity in the subcortical and periventricular white matter…” |
|  | Scattered | Mild | T505: “There are scattered foci and confluent areas of T2/FLAIR hyperintensity in the subcortical, deep and periventricular white matter as well as the central pons, a non-specific finding but likely reflecting the sequela of chronic microangiopathy.”  T571: “There are a few scattered foci of T2/FLAIR hyperintensity…”  T602: “There are mild scattered foci…” |
|  | Several | Moderate |  |
|  | Multiple | Moderate | T514: “There are multiple foci of T2/FLAIR hyperintensity…”  T581: “There are multiple scattered small foci and confluent areas of T2/FLAIR hyperintensity…” |
|  | Extensive | Severe | T613: “Extensive nonspecific bilateral T2/FLAIR hyperintensities…” |
|  | Diffuse | Severe | T597: “There are diffuse bilateral periventricular areas of T2/FLAIR hyperintensity…” |
|  | Mild-to-moderate, mild-moderate | Mild-moderate  (only use this category for this specific descriptor) |  |
|  | Moderate-to-severe, moderate-severe | Moderate-severe  (only use this category for this specific descriptor) |  |
| Topography/area of white matter hyperintensities (MRI) or hypodensities (CT) | Punctate | No influence on annotation | T528: “There are punctate and more confluent foci of T2/FLAIR hyperintensity in the periventricular and subcortical white matter.” |
|  | Focal, foci | No influence on annotation | T503: “There are additional foci of T2/FLAIR signal hyperintensity within the subcortical and periventricular white matter with additional involvement of the central pons, which are nonspecific although are presumably on the basis of chronic small vessel ischemic disease.” |
|  | Confluent | No influence on annotation | T521: “Focal and confluent subcortical and periventricular T2 and FLAIR hyperintensity is noted…”  T605: “Focal and confluent periventricular and subcortical white matter hypodensities are noted in the bilateral cerebral hemispheres, which likely represent mild changes of chronic small vessel ischemic disease.” |

**Expanded Methods: Neuroimaging direct review annotation guide**

Template hierarchy

1. Study ID
2. SBI
   1. Present
      1. No
      2. Yes
      3. Indeterminate
   2. If SBI indeterminate, most likely diagnosis?
      1. [Free text]
   3. Acuity
      1. acute/subacute
      2. chronic
      3. both
   4. Location: Cortical vs Subcortical (analogous to Location1 from Task 1)
      1. lacunar/subcortical
      2. cortical/juxtacortical
      3. both
   5. Location: Region (analogous to Location2 from Task 1)
      1. frontal
      2. parietal
      3. temporal
      4. occipital
      5. insular
      6. caudate
      7. putamen
      8. globus pallidus
      9. thalamus
      10. brainstem
      11. cerebellum
      12. more than one
      13. other
   6. Number
      1. one
      2. two
      3. three or more
3. WMD
   1. Present
      1. No
      2. Yes
      3. Indeterminate
   2. If WMD indeterminate, most likely diagnosis?
      1. [Free text]
   3. Grade (Manolio grading scale)
      1. 0
      2. 1
      3. 2
      4. 3
      5. 4
      6. 5
      7. 6
      8. 7
      9. 8
      10. 9
      11. 10
4. Comment – space for notes about cases, including mention of non-SBI, non-WMD findings

**Consensus Imaging Definitions**

*These imaging definitions are a composite of consensus definitions from the STRIVE consortium (endorsed by the AHA-ASA 2016 scientific statement on silent cerebrovascular diseases), definitions proposed in the grant application for this R01 funded project, and research team expertise. These definitions are broad guidelines for categorization of imaging findings potentially suggesting silent brain infarcts, white matter disease, and mimics.*

Study population:

- age > 50
- no prior clinical history or diagnosis of ischemic stroke, transient ischemic attack, or dementia
- neuroimaging requisition without indication for stroke, transient ischemic attack, dementia, or a focal neurologic symptom highly likely to be associated with stroke
- no prior clinical history or diagnosis of a condition potentially impeding the assessment of stroke prevention treatment effects (stroked unspecified; intracranial hemorrhage; traumatic brain injury; other symptomatic intracranial pathologies including anoxic brain injury, brain tumor, brain abscess of other intracranial infections potentially causing lasting neurologic deficits, multiple sclerosis)

**Positive imaging findings**

1. **Silent brain infarcts**

*Brain infarcts vary in chronicity, location, size, and several other imaging parameters. For the purposes of this study, the primary differentiating factors will be chronicity and cortical/subcortical location. Minimum size will primarily be relevant for chronic subcortical infarcts, whereas acute cortical or subcortical infarcts can be smaller than 3 mm in diameter.*

CT definition:

- **Subcortical**: Discrete, focal hypodense lesion ≥ 3 mm in size conforming to a vascular distribution in the white matter, grey matter, or both
- **Cortical/juxtacortical**: Discrete, focal hypodense lesion conforming to a vascular distribution in the grey matter or grey-white junction

MRI definition:

- **Acute/subacute, cortical/juxtacortical**: Discrete, focal, T2 hyperintense lesion conforming to a **vascular distribution** in the grey matter or grey-white junction **with restricted diffusion** (on DWI +/- ADC sequences)
- **Acute/subacute, subcortical**: Discrete, focal T2 hyperintense lesion conforming to a **vascular distribution** in the white matter, grey matter, or both **with restricted diffusion** (on DWI +/- ADC sequences)
- **Chronic, cortical/juxtacortical**: Discrete, focal, T2 hyperintense lesion conforming to a **vascular distribution** in the grey matter or grey-white junction **with at least one of the following: T1 hypointensity or volume loss**
- **Chronic, subcortical**: Discrete, focal T2 hyperintense lesion ≥ 3 mm in size conforming to a **vascular distribution** in the white matter, grey matter, or both **with at least one of the following: T1 hypointensity or cavitation with an irregular margin (i.e. ragged rim)**

|  |  | Acute/subacute, cortical | Acute/subacute, subcortical | Chronic, cortical | Chronic, subcortical |
| --- | --- | --- | --- | --- | --- |
| CT | Diameter |  |  |  | ≥ 3 mm |
|  | Density | Hypo | Hypo | Hypo | Hypo |
| MRI | Diameter |  |  |  | ≥ 3 mm |
|  | DWI | Hyperintense | Hyperintense | Isointense or Hypointense | Isointense or Hypointense |
|  | ADC | Hypointense (within 4-7 days) | Hypointense (within 4-7 days) | Hyperintense | Hyperintense |
|  | FLAIR | Hyperintense | Hyperintense | Hyperintense | Hyperintense |
|  | T2 | Hyperintense, no rim (homogeneous) | Hyperintense, no rim (homogeneous) | Hyperintense | Hyperintense, ragged rim |
|  | T1 | Hypointense | Hypointense | Hypointense or volume loss | Hypointense or cavitation |

2. **White matter disease/leukoaraiosis** (possible or probable vascular etiology)

CT definition: White matter lesions with low attenuation (compared to surrounding white matter), indistinct margins or confluent distribution, and not meeting criteria for brain infarction (i.e. no cavitation). Isolated lesions in the subcortical grey matter and brainstem are not included in this category.

MRI definition: White matter lesions with T2 hyperintensity, indistinct margins or confluent distribution, and not meeting criteria for brain infarction (i.e. no prominent T1 hypointensity, cavitation, or restricted diffusion). Isolated lesions in the subcortical grey matter and brainstem are not included in this category.

|  |  | Chronic, subcortical |
| --- | --- | --- |
| CT | Diameter |  |
|  | Density | Hypo |
| MRI | Diameter |  |
|  | DWI | Isointense or Hypointense |
|  | ADC | Hyperintense |
|  | FLAIR | Hyperintense |
|  | T2 | Hyperintense |
|  | T1 | Isointense (or minimally hypointense) |

**Examples of alternative imaging findings**

1. Perivascular spaces

|  |  | Chronic, subcortical |
| --- | --- | --- |
| CT | Diameter | Usually < 3 mm |
|  | Density | Hypo |
| MRI | Diameter | Usually < 3 mm |
|  | DWI | Hypointense |
|  | ADC | Hyperintense |
|  | FLAIR | Hypointense |
|  | T2 | Hyperintense, no rim |
|  | T1 | Hypointense |

2. Encephalomalacia (not due to ischemia; including trauma, post-surgical changes, degenerative brain atrophy, etc.)

|  |  | Chronic, cortical | Chronic, subcortical |
| --- | --- | --- | --- |
| CT | Diameter |  |  |
|  | Density | Hypo | Hypo |
| MRI | Diameter |  |  |
|  | DWI | Isointense or Hypointense | Hypointense |
|  | ADC | Hyperintense | Hyperintense |
|  | FLAIR | Hyperintense | Hypointense |
|  | T2 | Hyperintense, ragged rim | Hyperintense, no rim |
|  | T1 | Hypointense | Hypointense |

References:

1. Wardlaw JM, Smith EE, Biessels GJ, et al; STandards for ReportIng Vascular changes on nEuroimaging (STRIVE v1). Neuroimaging standards for research into small vessel disease and its contribution to ageing and neurodegeneration. Lancet Neurol. 2013;12:822–838.
2. Fanning JP, Wesley AJ, Wong AA, Fraser JF. Emerging spectra of silent brain infarction. Stroke. 2014;45:3461-3471.

**Supplemental Table 1. Interrater reliability for SBIs and WMD for RI and DR.**

|  | Characteristic | Cohen’s kappa or Spearman’s | 95% CI | p |
| --- | --- | --- | --- | --- |
| RI  (n = 400) |  |  |  |  |
|  | ± SBI | 0.88 | 0.80-0.97 |  |
|  | SBI acuity | 0.72 | 0.90-1.00 |  |
|  | SBI number | 0.89 |  | < 0.001 |
|  | SBI location | 0.36 | 0.59-0.83 |  |
|  | ± WMD | 0.98 | 0.97-1.00 |  |
|  | WMD grade | 0.99 |  | < 0.001 |
| DR  (n = 182) |  |  |  |  |
|  | ± SBI | 0.58 | 0.46-0.69 |  |
|  | SBI acuity | 0.66 | 0.3-1.00 |  |
|  | SBI number | 0.73 |  | < 0.001 |
|  | SBI location | 0.57 | 0.47-0.67 |  |
|  | ± WMD | 0.49 | 0.35-0.63 |  |
|  | WMD grade | 0.71 |  | < 0.001 |

RI = report interpretation, DR = direct review, SBIs = silent brain infarcts, WMD = white matter disease.

**Supplemental Table 2. Interrater Reliability for DR Across CT and MRI**

|  | CT | MRI |
| --- | --- | --- |
| SBI (n = 182) | 0.56 | 0.62 |
| WMD  (n = 182) | 0.41 | 0.47 |

**Supplemental Table 3. Interrater Reliability for DR Across Two Institutions**

| Comparison Type | Kappa |
| --- | --- |
| Intra-institution | 0.63 |
| Inter-institution | 0.57 |
